# Postpartum Headache: A Common Symptom for Different Diagnoses

**DOI:** 10.7759/cureus.108035

**Published:** 2026-04-30

**Authors:** Rui Macedo-Campos, Diana Silva, Carlos Silva, Joana Sousa Correia

**Affiliations:** 1 Anesthesiology, ULS (Unidade Local de Saúde) Santa Maria, Lisbon, PRT; 2 Anesthesiology, ULS (Unidade Local de Saúde) Médio Ave, Famalicão, PRT; 3 Anesthesiology, ULS (Unidade Local de Saúde) Barcelos Esposende, Barcelos, PRT

**Keywords:** differential diagnosis, labor epidural analgesia, neuraxial anesthesia complications, obstetric anesthesia, post-dural puncture headache (pdph), postpartum headache, puerperium

## Abstract

Headache is a common symptom in the postpartum period. The combination of postpartum headache and neuraxial analgesia or anaesthesia, even without evidence of dural puncture, may suggest post-dural puncture headache (PDPH), especially if there is a correlation between the headache and orthostatic position. However, there are many possible causes for postpartum headache. This report highlights the diagnostic challenge of postpartum headache in patients who received neuraxial analgesia, where an initial misleading diagnosis of PDPH delayed recognition of an alternative etiology. It underscores the importance of maintaining a broad differential diagnosis and performing a detailed clinical assessment of typical and associated symptoms to identify overlapping or non-PDPH causes.

## Introduction

Headache is one of the most common symptoms in the postpartum period, with up to 39% of parturients experiencing headache in the first postpartum week [1]. There are many possible causes of headache in the postpartum period. Almost 50% are tension type and migraine, preeclampsia and eclampsia are responsible for nearly a quarter, and post-dural puncture headache (PDPH) is the etiology in 16% of cases [1]. PDPH remains a prominent clinical concern for anesthesiologists. Despite being a benign condition, it can be poorly tolerated due to mobility limitations and challenges with newborn care.

Postpartum (puerperal) headache refers to headache occurring after delivery, while neuraxial anesthesia encompasses spinal and epidural techniques commonly used for labor analgesia. PDPH is a well-recognized complication of dural puncture, typically characterized by an orthostatic component [1].

Nonetheless, it is important to emphasize the need for anesthesiologists to consider different causes of postpartum headaches. Misdiagnosis of postpartum headache may delay recognition of serious underlying conditions and lead to inappropriate or delayed management, with potential implications for maternal safety and recovery during a critical period of newborn care [2]. Other important causes of postpartum headache include cerebral venous sinus thrombosis, intracranial hemorrhage, and infectious processes, such as meningitis, which require prompt recognition due to potential severity. Less commonly, musculoskeletal causes, such as cervicogenic headache, may also contribute to postpartum headache and can be easily overlooked, particularly in the presence of confounding features following neuraxial anesthesia [2-4].

We present a case of postpartum headache following neuraxial analgesia, in which an initial diagnosis of PDPH was reconsidered after further evaluation revealed a probable cervicogenic origin. The case is particularly relevant due to the diagnostic ambiguity created by overlapping clinical features, including transient improvement after an epidural blood patch, highlighting the risk of premature diagnostic closure in postpartum headache assessment.

## Case presentation

Obstetric and anesthetic course

A 36-year-old primigravid Caucasian woman at 39 weeks and 2 days of gestation was admitted in active labor to the obstetrics emergency department. Her medical history was unremarkable except for gestational diabetes. An epidural catheter was placed for pain relief at the L3-L4 intervertebral space. Through a midline approach and using a loss-of-resistance-to-saline technique, the epidural space was found at a distance of 5 cm from the skin, and 4 cm of an epidural catheter was introduced, indwelling the needle, without complications. Epidural analgesia was performed with ropivacaine 0.2% and sufentanil. Additional boluses of local anesthetic without opioids were administered for labour analgesia. Two hours later, a newborn male was born by vaginal delivery with an APGAR score of 9/10/10.

Clinical course and diagnostic reasoning

Twelve hours postpartum, the anesthetic team was called to evaluate the patient due to headache and cervical pain, which had begun immediately after delivery. The headache was initially accompanied by marked cervical stiffness, reduced range of motion, and significant pain exacerbation with head movement, to the extent that the patient required assistance for mobilization. Focused neurological examination revealed no focal deficits, with preserved cranial nerve function, normal strength and sensation, and no signs of altered mental status. Obstetric assessment showed no evidence of hypertensive disease, with normal blood pressure values and absence of proteinuria. No fever had occurred. Intravenous paracetamol, oral ibuprofen, diazepam, and local heat were prescribed, and a few hours later, cervicalgia showed improvement, and the puerpera was able to walk and feed herself. At this stage, marked cervical pain and reproducible exacerbation with neck movement were already present, which could suggest a significant musculoskeletal component.

On the second postpartum day, the patient reported worsening symptoms, with headache extending from the cervical region to the frontal area. The pain had a clear postural component, worsening on sitting or standing and improving in the supine position. In the absence of other neurological symptoms and in the context of recent neuraxial analgesia, a diagnosis of PDPH was considered the most likely explanation. Conservative management, including bed rest, hydration, caffeine, and analgesia, was initiated.

Despite conservative therapy, symptoms persisted over the following 72 hours. Given the persistence of a postural headache after neuraxial analgesia and in the absence of contraindications, an epidural blood patch was performed, as PDPH remained the leading diagnostic hypothesis despite the absence of a confirmed dural puncture. A total of 20 mL of autologous blood was injected into the epidural space without complications, resulting in significant symptomatic relief. The patient was discharged the following day with appropriate safety advice.

Twenty-four hours after discharge, the patient was readmitted to the emergency department due to recurrence of symptoms, including cervicalgia, neck stiffness, headache, and photophobia. On examination, she had a restricted cervical range of motion and mild lumbar discomfort, without signs of local infection at the puncture site. No focal neurological deficits were identified.

Vital signs were within normal limits, including stable blood pressure values (120-130 mmHg systolic), and there was no evidence of proteinuria. Laboratory tests showed mild leukocytosis (12,000/mm³), with only mild elevation of C-reactive protein (13 mg/L) and normal procalcitonin (<0.1 ng/mL), making both an infectious etiology and a hypertensive disorder of pregnancy unlikely.

**Figure 1:**
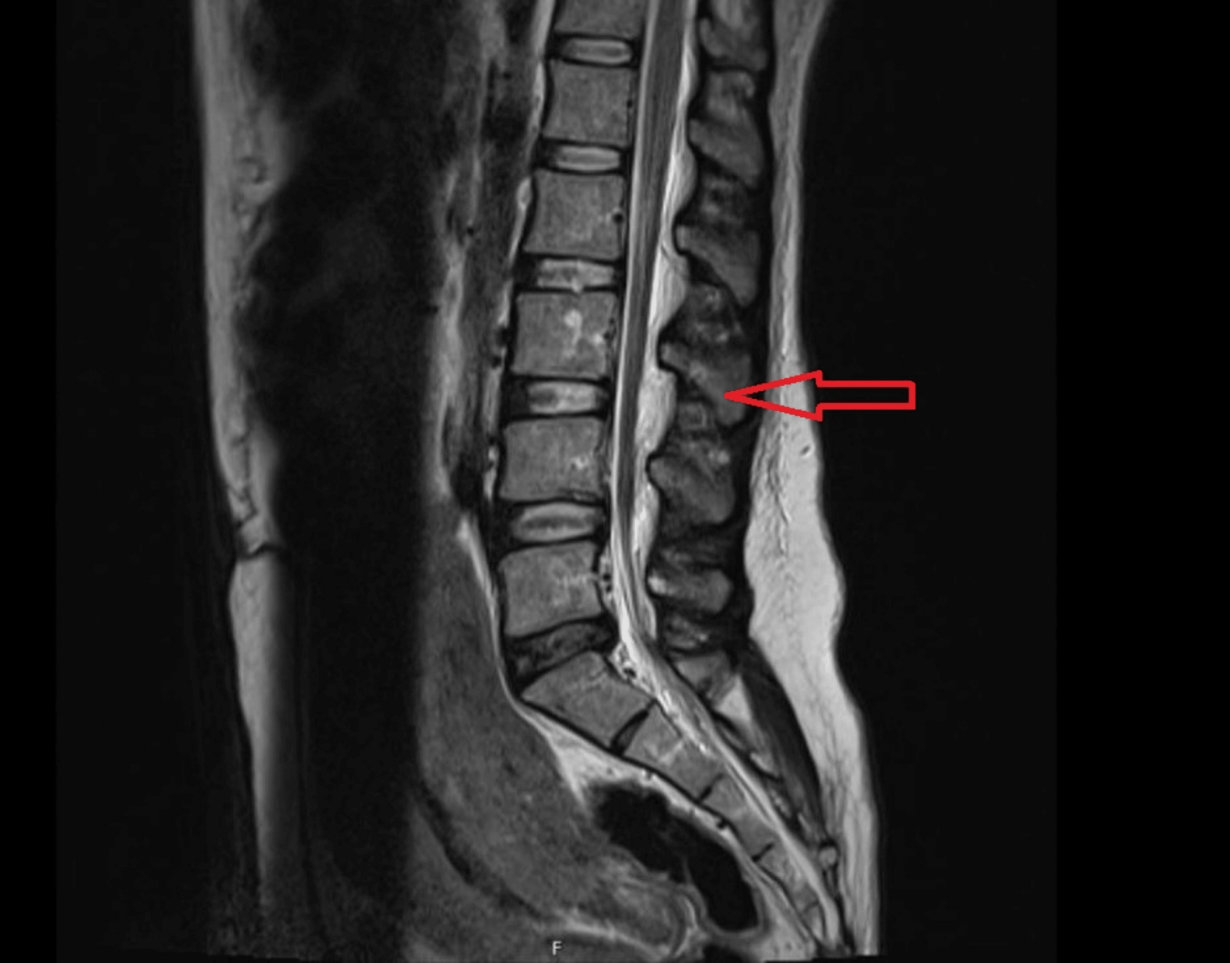
Lumbar spine magnetic resonance imaging The image demonstrates no evidence of a cerebrospinal fluid leak or dural sac discontinuity at the L3–L4 level (marked with an arrow), corresponding to the site of epidural catheter placement. No epidural or paravertebral fluid collections are identified, making a persistent cerebrospinal fluid fistula unlikely. Mild degenerative changes are noted at L5–S1, including a small left paramedian disc protrusion contacting the S1 nerve root, without clinical correlation to the presenting symptoms.

The patient reported partial symptom relief in the supine position and with intravenous paracetamol. Given the recurrence of symptoms after initial improvement with an epidural blood patch (EBP) and the persistence of diagnostic uncertainty, further clinical evaluation was pursued. The anesthetic team decided to request a lumbar magnetic resonance image (Figure 1). This imaging was primarily used to exclude persistent cerebrospinal fluid leakage or structural complications related to neuraxial anesthesia. In the absence of red flag symptoms, such as focal neurological deficits, persistent pain refractory to analgesia, signs of increased intracranial pressure, or altered mental status, the suspicion of an intracranial pathology was considered low, and no further neuroimaging was deemed necessary at that stage.

During reassessment, additional clinical history revealed a previous follow-up in Neurology for cervicogenic headache, which had not been initially disclosed. Neurological evaluation documented bilateral cervico-occipital pain triggered and exacerbated by neck movement, associated with restricted cervical range of motion, muscular contracture, and crepitation in the cervico-occipital region, reported to have developed following expulsive efforts during labor. No cervical spine imaging was performed.

Based on the clinical presentation and according to features described in the International Classification of Headache Disorders (ICHD)-3 criteria for cervicogenic headache [3], namely, the temporal relationship between cervical symptoms and headache onset, reduced cervical mobility, and worsening with provocative neck movements, a diagnosis of probable cervicogenic headache was established. Targeted treatment with diclofenac, diazepam, and local moist heat was initiated, with subsequent clinical improvement. Two days later, the puerpera was discharged home with outpatient Neurology follow-up.

The initial diagnosis of PDPH was primarily based on the temporal association with neuraxial analgesia and the presence of a postural headache. However, features that should have prompted earlier reconsideration included the prominence of cervical pain, reproducibility of symptoms with neck movement, and incomplete response to targeted analgesia despite typical PDPH management. These findings became more evident during reassessment and, together with the previously unreported history of cervicogenic headache, shifted the diagnostic interpretation. Table 1 summarizes the key differential diagnoses to consider in postpartum headache following neuraxial anesthesia, along with their distinguishing clinical features.

**Table 1:** Differential diagnosis of postpartum headache after neuraxial anesthesia PDPH: post-dural puncture headache; CSF: cerebrospinal fluid; CRP: C-reactive protein

Diagnosis	Key features	Present case
PDPH	Orthostatic headache, post neuraxial, CSF leak	Initially suspected
Cervicogenic headache	Neck pain, movement-related, muscular trigger	Ultimately diagnosed
Preeclampsia	Hypertension, proteinuria	Excluded
Cerebral venous sinus thrombosis	Focal deficits, severe headache	No red flags
Meningitis	Fever, neck rigidity, raised CRP	Unlikely

## Discussion

There are numerous causes of postpartum headache, including vascular, infectious, neoplastic, pharmacological, metabolic, and musculoskeletal conditions, as well as primary headache disorders and PDPH [1,5]. PDPH is a strong possibility in the clinical context of neuraxial anesthesia; however, a complete history and physical examination should be performed, considering the timing of the headache in relation to the neuraxial procedure, the nature of the headache, as well as other associated symptoms and signs [5-7].

In this report, a headache accompanied by cervical pain, which improved in the supine position, in a woman who received neuraxial anesthesia, led the anesthetic team to assume a scenario of PDPH. Conservative treatment followed by an epidural blood patch was performed. However, after an initial improvement, the patient was readmitted to the emergency department with recurrence of symptoms. After hospital readmission, imaging studies were performed, and the neurology team carried out a more detailed characterization of the headache, describing pain in the cervico-occipital region elicited by palpation and mobilization of the neck, which was associated with a cervical muscular contracture. A diagnosis of a possible cervicogenic headache was established, and medical treatment with local moist heat, anti-inflammatories, and benzodiazepines was instituted. The previous diagnosis of PDPH was therefore less likely, or alternatively, a combination of two clinical entities may have occurred, contributing to the onset of headaches and the associated symptoms.

In the assessment of any parturient with a postpartum headache, it is important to carefully consider the differential diagnosis and red flag signs that may warrant immediate neuroimaging studies. The majority of headaches in this period are tension-type headaches and migraines [1]. However, the combination of pregnancy, labor, neuraxial anesthesia or analgesia, and postpartum headache with a typical orthostatic component may suggest PDPH, even without evidence of a dural puncture during the epidural technique [6]. The risk of unintentional dural puncture with a Tuohy needle during epidural anesthesia is 1.5%, and half of these patients will experience PDPH [7]. Up to 38% of PDPH cases can arise after a seemingly uneventful procedure [8]. PDPH usually presents as a throbbing or dull fronto-occipital headache, with a typical postural component, usually but not invariably orthostatic, improving with the supine position [1,6]. It is likely to be caused by low cerebrospinal fluid pressure with traction on intracranial structures or compensatory cerebral vasodilatation and is usually accompanied by neck stiffness, dizziness, nausea and vomiting, visual disturbances, photophobia, or hearing symptoms. PDPH normally occurs in the first 72h, but it can appear within 5 days after dural puncture [1,6].

Conservative measures include bed rest, hydration, simple analgesics, and caffeine. When conservative management fails, a sphenopalatine block or an EBP can be performed. In 40% of the cases, a second procedure is required, despite initial pain relief [9]. Nevertheless, these techniques are not free of risks, and complications related to an EBP can occur such as meningitis, subdural haematoma, arachnoiditis, or radicular pain [1].

Despite the above being a common cause of postpartum headache, other diagnoses should be excluded, especially when there are other associated symptoms. In the evaluation of postpartum headache, a structured differential diagnosis is essential [2]. In this case, PDPH was initially considered, given the temporal relationship with neuraxial analgesia and the presence of orthostatic features. However, other important conditions were also considered. Hypertensive disorders, such as preeclampsia, were deemed unlikely given normal blood pressure values and absence of proteinuria. Infectious causes, including meningitis, were considered less probable in the absence of fever, meningeal signs, or significant inflammatory markers. Although cranial neuroimaging was not performed, the absence of focal neurological deficits or other red flag symptoms made intracranial hemorrhage or cerebral venous sinus thrombosis less likely, though not definitively excluded. The subsequent identification of a prior history of cervicogenic headache, together with the presence of cervico-occipital pain triggered by neck movement and associated with muscular contracture, strongly supported this diagnosis.

Cervicogenic headache is causally associated with myofascial pain sources or trigger points. It is caused by a disorder of the cervical spine and its components (bone, disc, and soft tissue), and it is usually but not invariably accompanied by neck pain [6]. In this case, prolonged expulsive efforts during labor may have contributed to cervical muscular strain and symptom development. Features that tend to distinguish it from migraine and tension type headache include side-locked pain, headache with digital pressure of neck muscles and head movement, and posterior-to-anterior radiation of pain [6].

It is not uncommon for anesthetists to be called to evaluate a puerpera with a headache in the postpartum period. In this report, while the possibility of an unrecognized dural puncture cannot be definitively excluded, especially in view of the transient improvement following the EBP, a subsequent thorough clinical examination revealed cervical muscle contracture, with symptoms being elicited and exacerbated by manipulation and pressure of cervical trigger points.

The transient improvement following an EBP raises relevant diagnostic considerations. This response may reflect a placebo effect, a partial contribution of an unrecognized low-pressure component, or the coexistence of overlapping pathologies. Although the initial clinical presentation, including orthostatic features and temporal association with neuraxial analgesia, favored PDPH, subsequent evolution and the absence of imaging evidence of cerebrospinal fluid leak made a purely low-pressure etiology less likely. A mixed mechanism, with partial response of a potential PDPH component and concomitant resolution of a cervicogenic pain syndrome, remains a plausible explanation.

This case highlights an important diagnostic pitfall in the evaluation of postpartum headache after neuraxial analgesia. The combination of orthostatic headache, cervical pain, and transient improvement after an EBP initially strongly supported PDPH, contributing to diagnostic anchoring. However, reassessment revealed features more consistent with a probable cervicogenic headache, including pain exacerbation with neck movement, cervical muscular contracture, and a previously unreported history of similar symptoms under Neurology follow-up.

Although PDPH is frequently considered the leading diagnosis in postpartum patients who underwent neuraxial procedures, the study by Goldszmidt et al. demonstrated that PDPH accounts for only a minority of postpartum headaches, even in the presence of postural symptoms [3]. Similar diagnostic challenges have been described in previous reports, emphasizing that orthostatic features and even transient response to EBP are not entirely specific for PDPH [4]. Other primary and secondary causes of headache may mimic PDPH or coexist simultaneously, while publication bias may contribute to an overrepresentation of more severe causes of postpartum headache in the literature [2,3]. To our knowledge, reports describing overlap between cervicogenic headache and suspected PDPH in the postpartum setting remain exceedingly rare.

This case emphasizes that postpartum headache after neuraxial anesthesia should not be attributed automatically to PDPH, even in the presence of orthostatic features or a transient response to an EBP. Cervicogenic headache, although less common in this setting, may mimic PDPH and contribute to diagnostic uncertainty. Repeated structured reassessment, including careful musculoskeletal examination and review of previous headache history, is essential to avoid premature diagnostic closure.

## Conclusions

This case illustrates the diagnostic complexity of a postpartum headache following neuraxial anesthesia, particularly when overlapping clinical features suggest PDPH. Although the patient initially presented with orthostatic headache and transient improvement after an EBP, subsequent reassessment revealed findings more consistent with a probable cervicogenic headache, while the coexistence of both entities could not be completely excluded.

While conclusions from a single observational case should be interpreted cautiously, this report suggests that postpartum headaches after neuraxial procedures may not always be exclusively attributable to PDPH, even in the presence of typical orthostatic features. Repeated clinical reassessment, careful symptom characterization, and evaluation of associated signs may help clinicians identify alternative or overlapping diagnoses and avoid premature diagnostic closure.

## References

[1] Sabharwal A, Stocks GM (2011). Postpartum headache: diagnosis and management. Contin Educ Anaesth Crit Care Pain.

[2] Klein AM, Loder E (2010). Postpartum headache. Int J Obstet Anesth.

[3] Goldszmidt E, Kern R, Chaput A, Macarthur A (2005). The incidence and etiology of postpartum headaches: a prospective cohort study. Can J Anaesth.

[4] Di Paolo M, Maiese A, Mangiacasale O (2021). Don’t forget rare causes of postpartum headache! Cases report and literature review. Medicina (Kaunas).

[5] Campbell NJ (2026). Effective management of the post dural puncture headache. Anesthesia tutorial of the week. 181. 31st May 2010. Anesthesia Tutoral of the Week, World Federation of Anesthesia.

[6] Headache Classification Committee of the International Headache Society (IHS) (2013). The International Classification of Headache Disorders, 3rd edition (beta version). Cephalalgia.

[7] Choi PT, Galinski SE, Takeuchi L, Lucas S, Tamayo C, Jadad AR (2003). PDPH is a common complication of neuraxial blockade in parturients: a meta-analysis of obstetrical studies. Can J Anaesth.

[8] Van de Velde M, Schepers R, Berends N, Vandermeersch E, De Buck F (2008). Ten years of experience with accidental dural puncture and post-dural puncture headache in a tertiary obstetric anaesthesia department. Int J Obstet Anesth.

[9] Paech M (2005). Epidural blood patch-myths and legends. Can J Anaesth.

